# Estimation of genetic polymorphism in quince (*Cydonia oblonga* Mill.) genotypes using morphological traits and molecular (DNA barcoding) characterizations

**DOI:** 10.1371/journal.pone.0310048

**Published:** 2024-11-13

**Authors:** Syed Hassan Ali Shah, Mohammad Nisar, Mohammad Ihsan, Muhammad Zahoor, Riaz Ullah, Zafar Iqbal, Abdul Bari Shah

**Affiliations:** 1 Department of Botany, University of Malakand, Dir Lower, Pakistan; 2 Department of Biochemistry, University of Malakand, Dir Lower, Pakistan; 3 Department of Pharmacognosy, College of Pharmacy, King Saud University, Riyadh, Saudi Arabia; 4 Department of Surgery, College of Medicine, King Saud University, Riyadh, Kingdom of Saudi Arabia; 5 Natural Products Research Institute, College of Pharmacy, Seoul National University, Seoul, Republic of Korea; Government Degree College Totakan, PAKISTAN

## Abstract

Quince (*Cydonia oblonga*) is a medicinal plant and a member of family Rosaceae. It is native plant of Asia Minor and Europe. It is used in production of jam and jellies and also as a remedy of several ailments. The present study was conducted to evaluate the genetic polymorphism based on morphological and molecular traits. Different varieties of Quince were collected from different ecological zones of Khyber Pakhtunkhwa Pakistan and a total of 26 different morphological traits were recorded among studied genotypes. Based on qualitative morphological trait study, the variety collected from Tindodag was unique one with highest fruit weight (328.82 g). The lowest fruit weight (68.38 g) was recorded for Talash genotype. The Charbagh and Tindodag genotypes showed highest seed length (10.6 mm) while genotypes of Chitral was recorded as lowest (8.4 mm). Statistically, significant level of variation was noted with coefficient of variance ranged from 2.23% to 30.38%. Based on correlation analysis, fruit length had strongly correlation with fruit weight (r = 0.89**), Average Fruit width was found significant with fruit weight (r = 0.90**). Similarly, the Core Width was found strongly significant with Core Length (r = 0.95**). ANOVA analysis indicated 10 quantitative characters to be highly significant, 2 significant and 1 insignificant. Principal component analysis was also computed for the 13 quantitative traits with Eigen value of 0.48 and a total variance of 97.78%. The first principal component shows total variation of 52.52%. In PC2 the total variation was 80.15%, PC3 94.06% while in PC4 it was 97.78%. The NCBI BLAST results shows that all the genotypes have similar origin except Tindodag genotype, which shows differences in its origin. Accession number for all other genotypes is MN216014.1, while accession number of Tindodag genotype is KF861967.1. Based on this study, it can be concluded that Tindodag genotype is unique out of the studied localities. NCBI BLAST have provided further support for the drawn conclusion.

## Introduction

Quince (*Cydonia oblonga* Mill.) is an important medicinal plant belonging to the family of Rosaceae [1]. It is medicinal and is used to treat several ailments like hepatitis, cancer and diabetes [2]. It is a shrub or a small tree approximately 8 meters tall [3]. Branchlets, when young, are covered with light greyish wool. This Plant has elliptical leaves and flowers are white or pink in color. Fruit is bright yellowish when ripe [4] and is rich in flavor and sweet aroma [5]. Fruit has large number of mucilage-coated, plano-convex seeds. The seeds are tightly wrapped in two vertical rows [6].

*Cydonia oblonga* is the sole member of genus *Cydonia* [7]. There are two subspecies of *Cydonia oblonga*, one is *Cydonia oblonga* subsp. *Pyriformis*, which has pear shaped fruit, while the other is *Cydonia oblonga* subsp. *Maliformis*, which has apple shaped fruit [8]. Quince has more than 70 genotypes distributed around the globe. Pineapple, Spahan, Botermo, Ekmek, Meeh, Portugal, Champion are some of the most popular varieties [2]. Plant morphological assessment is one of the first steps in identifying genetic makeup of a plant. This method is simple, easy and reasonable in providing an overview of the given germplasm. Assessment of morphological diversity of quince is difficult because of close similarity of its fruit and leaves [9] that is why less research has been carried out on this plant. Tree growers have made some attempts to classify quince and give a name to the cultivar based on its shape [10]. Kafkas *et al*., [11] started collection of quince germplasm in different regions of Iran and collected almost 50 different genotypes of it. Five genotypes of quince were studied in Spain and their morphological, chemical and organoleptic characteristics were evaluated. Their evaluation showed uniformity in shape of fruit and leaf [10].

Environmental influences on phenotypes make it difficult to distinguish different cultivars. Different agronomic traits have been used as a prime objective to evolve new breeding material [11]. In order to experimentally characterize and identify a collection of germplasm, use of molecular markers is a reliable approach. Plant and fruit morphological properties of quince genotypes are similar to each other and it is difficult to differentiate closely related genotypes as mentioned above [12]. DNA barcoding was proposed by Herbert in 2003 as a tool of identify species [13]. DNA barcoding is considered a powerful tool in discovering and identifying species. One or more standardized short DNA regions are utilized in barcoding to identify a taxon [14]. Generally, for evolutionary genetics, mitochondrial DNA is a reliable marker because it lacks genetic recombination, high copy number and has rapid evolutionary rate [15]. Barcoding tends to solve limitations in the traditional identification process of species. It has become new tool for taxonomist to complement their knowledge. It is an innovative device for newbies, who want to quickly identify an organism [13].

Now a day, due to awareness of the medicinal values of quince as well as its commercial uses in jams and as rootstock, the plant has gained more attention in Khyber Pakhtunkhwa and other parts of the world. Hence forward, the study was planned to evaluate the morphological diversity and DNA-barcoding with conventional PCR and DNA sequencing to find out best genotype for further cultivation in the selected area to fulfill the demands of the local population.

## Material and methods

### Plant collection

The study was conducted at Department of Botany, University of Malakand KPK Pakistan. The Quince (*Cydonia oblonga* Mill) genotypes were collected from seven different locations of Khyber Pakhtunkhwa, Pakistan as listed in Table 1. A voucher specimen was deposited in the Botanical Garden and Herbarium University of Malakand (UOM.Bot23/Garden 706).

**Table 1:** Passport information of Pakistani quince landraces used in the present study.

Genotype	Origin	Country	Latitude	Longitude	Altitude
Charbagh	Swat, KPK	Pakistan	34.8333° N	72.4383° E	980 m
Talash	Dir L, KPK	Pakistan	34.86° N	71.79° E	898 m
Chitral	Chitral, KPK	Pakistan	35.8540° N	71.7866° E	1494 m
Tindodag	Swat, KPK	Pakistan	34.7486° N	72.2897° E	666 m
Dardyal	Swat, KPK	Pakistan	34.9358° N	72.2073° E	1136 m
Nalkot	Swat, KPK	Pakistan	35.0644° N	72.3067° E	1476 m
Malam Jabba	Swat, KPK	Pakistan	34.7993° N	72.5714° E	2804 m

### Morphological characterizations

A number of exploratory trips were arranged to various agro-ecological zone of Khyber Pakhtunkhwa Pakistan to collect data about different varieties Quince. The Plant material (Quince landraces) was identified by expert, and data were recorded manually. Five plants from each area/site were randomly selected and the mean value was used while analyzing the collected data. The morphological data were recorded using the method of *Monka et al*., [1], a total of 26 morphological traits were targeted (both quantitative and qualitative). Out of these 26 parameters, 13 were qualitative traits and 13 were quantitative traits. Qualitative characters included plant vigor, plant growth habit, leaf time, flowering time, leaf color, plant Core length, fruit density, fruit size and fruit shape, along with symmetry in longitudinal section, neck size, ripening time, and fruit taste. Among the 13 Quantitative characters, whole fruit weight, exocarp weight, mesocarp weight, seed weight, fruit length, average fruit width 10 mm above basal part and 10 mm below apical part, core length, core width, average middle fruit width, seed width, seed length, and number of seeds per fruit.

### Morphological data analysis

As mentioned, both quantitative and qualitative traits were recorded from seven different experimental locations. The data were collected about both fruit and tree. Five plants were randomly selected from each genotype in their respective zone and their mean value was used for data analysis. Microsoft Excel 2016 was used to analyze the frequency distribution of qualitative traits. Recorded quantitative data was averaged and mean values were used for descriptive statistics, Correlation analysis using SPSS version 22, and Principal Component analysis was calculated using PC-ORD version 5.

### Mitochondrial DNA isolation

Mitochondrial DNA was isolated from Quince fresh leaves using the protocol of Nisar *et al*., [13]. Primers used in the study was designed following the procedure of Arleo *et al*., [14] as given Table 2. Nyx Technik thermal cycler was used to carry out primer amplification. PCR amplification was perform using procedure of Arif *et al*., [15]. After that, for commercial sequencing, they were sent to BGI Tech Solutions (Hong Kong) CO., Limited Hong Kong. The sequences were interpreted based on chromatogram and BLAST result. Then, with a reference database, it was searched using the NCBI BLAST (https://blast.ncbi.nlm.nih.gov/Blast.cgi). The sequence was then computed in order to assign them identity. The ID of each genotype was associated with the best BLAST hit and E-value cut off. This is in corresponding with the top hit in the BLAST result (BLAST1).

**Table 2:** Primers used in the present study of quince landraces for PCR.

Regions	Primers	Sequence	BP
matK	matK AF	5´-TCTTACCGAATAGGTCCAAAAC-3´	88
	matK AR	5´-GGCTCTACCCATTTATTCACTC- 3´	
matK	matK BF	5´-CTTCTCATTTACGATTAACC-3´	129
	matK BR	5´-CCTTGAAGAACCATAAGATG-3´	

## Results

### Morphological characterization

#### Diversity in qualitative traits

Different qualitative traits were recorded based on tree and fruit morphology, where a significant level of variation was observed. The data is presented in Table 3. The study shows that plant growth vigor was medium to vigorous in all the selected areas. Plant growth habit is semi upright and upright from place to place. Leaf time is from March to April end in different areas. Flower timing is in the month of March in all areas, starts from first week of March till the end of the month. Leaf color is either light green, or green or dark green depending on the locality. Plant height is recorded from 5–7 feet up to more than 10 feet in different areas. In most of the places, fruit density was medium, except Tindodag where it was dense and Malam Jaba where density was low.

**Table 3:** Diversity in qualitative traits of tree and fruit of quince genotypes collected from different ecological zones using *Monka et al*., 2014.

Tree morphology							
Genotypes	Plant vigor	Growth habit	Leaf time	Flowering time	Leaf color	Plant height	Fruit density
Charbagh	Non- vigorous	Upright	March end	March beginning	Light green	Tall	Low
Talash	Medium vigorous	Semi Upright	April	March	Light green	Tall	Medium
Chitral	Vigorous	Semi Upright	March end	March beginning	Medium Green	Tall	Medium
Tindodag	Vigorous	Upright	March end	March beginning	Dark green	Dwarf	Dense
Dardyal	Medium vigorous	Semi Upright	March to April	March beginning	Dark green	Dwarf	Medium
Nalkot	Medium vigorous	Semi Upright	March	March beginning	Dark green	Tall	Medium
Malam jaba	Medium vigorous	Upright	April	March End	Medium green	Dwarf	Low
Fruit Morphology							
Genotypes	Fruit Size	Fruit Shape	Symmetry in longitudinal section	Neck Size	Ripening Time	Fruit Taste	
Charbagh	Medium	Maliformis	Symmetric	Small	September	Astringic	
Talash	Small	Maliformis	Symmetric	Small	September	Astringic	
Chitral	Medium	Pyriformis	Symmetric	Medium	September	Astringic	
Tindodag	Large	Maliformis	Asymetric	Small	September	Edible	
Dardyal	Medium	Maliformis	Symmetric	Small	August End	Astringic	
Nalkot	Small	Maliformis	Asymetric	Small	October	Astringic	
Malam jaba	Small	Maliformis	Asymetric	Small	Ocober	Astringic	

Fruit morphology also shows significant level of diversity (Table 3). It is evident that fruit size is recorded from small to medium in different areas, while in Tindodag large fruit size is noted. Fruit shape in all other areas is maliformis (Apple shaped), except Chitral where fruit shape is pyriformis (Pear shaped). Longitudinal section of fruit shows symmetric to asymmetric shape. Neck size in Chitral fruit is medium, in all other places it is small. Ripening time is from August end to November. Fruit taste of all areas is astringent while Tindodag fruit variety is edible.

Variation is noted in morphological characters of both tree and fruit, but most of these variations are not that much note-worthy because they are either small or they may be due to ecological distribution, because temperature and altitude also greatly impact plant morphological characters. Two characters which indicates difference in genotype is reported from fruit. One is fruit taste, the genotype of Tindodag is distinct in this regard as it is edible. The other character is fruit shape, Fruit of Chitral genotype is noted to be Pyriformis in shape, which is distinct from all other genotypes.

### Diversity in quantitative traits

The descriptive statistics (mean, maximum, minimum, and CV %) of different quantitative traits recorded in the present study are presented in Table 4. Overall, significant diversity was observe for most of the traits and the coefficient of variation percent (CV%) ranged from 2.23 to 30.38% observed (Table 4). In quantitative morphology of the selected genotypes of quince, we determined average weight of the whole fruit, the mean shows that highest weight (328.82 g) was recorded in genotype collected from Tindodag, while lowest mean weight (68.38 g) was recorded in Talash genotype. Highest average exocarp weight (5.16 g) was also recorded in Tindodag genotype, while lowest average exocarp weight (2.49 g) was recorded in Malamjaba genotype. Genotype collected from Tindodag shows highest average mesocarp weight (320.41 g), while Talash genotype shows lowest value (44.48 g). Genotype collected from Tindodag shows highest average seed weight (4.02 gm), while genotype collected from Nalkot shows lowest average seed weight with the value of 1.04 g (Table 4).

**Table 4:** Diversity in quantitative traits of quince genotypes collected from different ecological zones.

Parameters	Sample	Variety	Minimum	Maximum	Mean	CV%
**Whole Fruit Weight (gm)**	Charbagh	Maliformis	157.42	181.21	169.52	6.09
	Talash	Maliformis	67.8	70.41	68.38	2.23
	Chitral	Pyriformis	195.2	250.1	225.63	9.94
	Tindodag	Maliformis	296.22	357.65	328.82	7.63
	Dardyal	Maliformis	179.9	201.12	188.10	4.48
	Nalkot	Maliformis	160.42	198.12	182.30	8.81
	Malam jaba	Maliformis	143.1	159.21	152.98	5.14
**Exocarp (gm)**	Charbagh	Maliformis	2.67	4.18	3.33	19.29
	Talash	Maliformis	2.16	3.42	2.76	19.39
	Chitral	Pyriformis	2.92	3.24	3.18	5.44
	Tindodag	Maliformis	4.82	5.62	5.16	6.17
	Dardyal	Maliformis	2.63	3.62	3.09	12.38
	Nalkot	Maliformis	2.42	3.11	2.68	10.44
	Malam jaba	Maliformis	2.13	3.15	2.49	16.41
**Mesocarp (gm)**	Charbagh	Maliformis	84.31	94.61	90.05	4.42
	Talash	Maliformis	40.1	48.63	44.48	7.33
	Chitral	Pyriformis	194.3	250.12	222.81	11.6
	Tindodag	Maliformis	290.06	345.24	320.41	7.31
	Dardyal	Maliformis	182.34	196.21	188.1	2.99
	Nalkot	Maliformis	162.39	184.21	176.41	5.07
	Malam jaba	Maliformis	138.21	158.25	147.48	5.15
**Seeds weight (gm)**	Charbagh	Maliformis	2.44	3.12	2.67	13.9
	Talash	Maliformis	2.56	3.54	2.98	12.57
	Chitral	Pyriformis	0.75	1.54	1.06	30.38
	Tindodag	Maliformis	3.87	4.12	4.01	2.86
	Dardyal	Maliformis	1.31	1.58	1.46	9.32
	Nalkot	Maliformis	0.82	1.32	1.04	21.22
	Malam jaba	Maliformis	1.98	2.42	1.28	7.85
**Fruit Length (mm)**	Charbagh	Maliformis	58.4	64.3	60.64	4.21
	Talash	Maliformis	38.2	44.5	41.24	5.94
	Chitral	Pyriformis	75.6	82.1	78.92	3.52
	Tindodag	Maliformis	78.1	86.4	81.9	4.3
	Dardyal	Maliformis	48.7	63.1	54.64	11.22
	Nalkot	Maliformis	44.6	54.2	50.28	7.65
	Malam jaba	Maliformis	44.1	56.2	49.48	9.9
**Core Length**	Charbagh	Maliformis	25.4	30.7	28.72	9.3
	Talash	Maliformis	19.3	24.4	20.86	12.54
	Chitral	Pyriformis	21.2	28.2	24.16	12.65
	Tindodag	Maliformis	21.3	26.3	23.58	8.72
	Dardyal	Maliformis	20.1	24.6	22.96	8.29
	Nalkot	Maliformis	20.3	24.6	22.12	8.15
	Malam jaba	Maliformis	18.7	24.2	21.36	10.98
**Core Width**	Charbagh	Maliformis	24.3	28.2	26.34	6.12
	Talash	Maliformis	18.6	23.3	20.32	10.92
	Chitral	Pyriformis	20	26.2	22.08	13.46
	Tindodag	Maliformis	20.9	25.2	22.96	9.81
	Dardyal	Maliformis	19.5	22.8	20.5	6.4
	Nalkot	Maliformis	18	23.6	21.36	10.4
	Malam jaba	Maliformis	17.8	23.9	20.54	11.08
**Average Fruit width (10mm below apical part (mm)**	Charbagh	Maliformis	51.5	58.7	54.58	5.34
	Talash	Maliformis	33.2	40.1	36.82	7.05
	Chitral	Pyriformis	71.6	76.7	73.64	2.93
	Tindodag	Maliformis	72.6	81.8	77.2	5.01
	Dardyal	Maliformis	44	58.1	49.74	12.94
	Nalkot	Maliformis	39.2	48	44.22	7.79
	Malam jaba	Maliformis	39	50.3	44.34	10.2
**Average fruit width (10mm above basal part (mm)**	Charbagh	Maliformis	48.2	54.3	51.24	4.5
	Talash	Maliformis	29.9	35.7	33.04	7.44
	Chitral	Pyriformis	68.4	74	71.68	3.18
	Tindodag	Maliformis	69.7	78.5	74.76	5.91
	Dardyal	Maliformis	42.2	57.3	47.96	13.01
	Nalkot	Maliformis	39.3	48.4	44.14	8.51
	Malam jaba	Maliformis	37.4	44.8	44.02	11.08
**Average middle fruit width (mm)**	Charbagh	Maliformis	60.72	68.5	65.82	4.75
	Talash	Maliformis	46.2	50.8	48.08	3.58
	Chitral	Pyriformis	72.8	77.1	74.86	2.38
	Tindodag	Maliformis	84.2	96.2	91.86	5.42
	Dardyal	Maliformis	48.2	63.5	57.24	10.39
	Nalkot	Maliformis	54.3	66.7	61.54	8.46
	Malam jaba	Maliformis	54.3	65.3	60.34	6.65
**Seed Length (mm)**	Charbagh	Maliformis	9	12	10.4	10.96
	Talash	Maliformis	9	12	10.6	10.75
	Chitral	Pyriformis	7	9	8.4	10.64
	Tindodag	Maliformis	9	12	10.6	14.3
	Dardyal	Maliformis	8	11	9.6	11.87
	Nalkot	Maliformis	8	10	9.2	9.09
	Malam jaba	Maliformis	8	10	9.4	9.51
**Seed Width (mm)**	Charbagh	Maliformis	4	6	4.8	17.43
	Talash	Maliformis	4	5	4.2	10.64
	Chitral	Pyriformis	3	4	3.2	13.97
	Tindodag	Maliformis	3	6	4.6	24.78
	Dardyal	Maliformis	4	5	4.4	12.44
	Nalkot	Maliformis	3	5	4	17.67
	Malam jaba	Maliformis	4	5	4.4	12.44
**number of seeds/fruit**	Charbagh	Maliformis	30	42	36.8	12.36
	Talash	Maliformis	43	60	52.8	13.08
	Chitral	Pyriformis	12	22	15.2	26.06
	Tindodag	Maliformis	49	65	58	10.55
	Dardyal	Maliformis	18	25	20.8	13.34
	Nalkot	Maliformis	8	17	12.6	28.94
	Malam jaba	Maliformis	51	66	58.4	10.31

Fruit length of the collected genotypes was evaluated, Tindodag genotype shows highest average value (81.9 mm), while Talash genotype shows lowest value (41.24 mm). Data shows highest core length value (28.72 mm) for Charbagh genotype and lowest value for Talash genotype (20.86 mm). Highest average of core width (26.34 mm) is also recorded for Charbagh genotype and lowest value (20.32 mm) for Talash genotype. Fruit was cut into two equal halves and its width was measured at three different points. Average width, at a point 10 mm below the neck of the fruit, was measured highest for Tindodag genotype (76.2 mm) and lowest for Talash genotype (36.82 mm). Average width 10 mm above the basal point was measured highest for Tindodag genotype (74.76 mm) and lowest for Talash genotype (33.04 mm). Genotype collected from Tindodag shows has highest average middle fruit width (91.86 mm) and collections from Talash shows lowest average width (48.08 mm) as tabulated in the Table 4. Charbagh and Tindodag genotypes show highest seed length (10.6 mm) while genotypes of Chitral recorded lowest seed length (8.4 mm). seed width is recorded highest for Charbagh genotype (4.8 mm) and lowest for Chitral genotype (3.2 mm). Highest average number of seeds per fruit are counted for Malam Jaba genotype (58.4) and lowest are counted for Nalkot genotype (12.6).

### Correlation analysis

The correlation analysis for different morphological traits are as presented in Table 5. The results indicate that fruit length is strongly correlated with whole fruit weight (*r* = 0.89**). 10 mm above basal part width is strongly correlated with fruit length (*r* = .0.99**). 10 mm below the apical part width has strong correlation with 10 mm above the basal part (*r* = 0.99**). Core length is significantly correlated with 10 mm below the apical part (*r =* 0.42). Core width is strongly correlated with core length (*r* = 0.95**). Exocarp weight is significantly correlated with Core width (*r* = 0.41). Middle fruit length is strongly correlated with exocarp weight (*r* = 0.86*). Seed weight is significantly correlated to Middle fruit length (*r* = 0.36). Seed length is strongly correlated with Seed weight (*r* = 0.91**). Seed width is strongly correlated with Seed length (*r* = 0.87**). Seeds per fruit is strongly correlated with Seed width (*r* = 0.64). Mesocarp weight is negatively correlated Seeds per fruit (*r* = -0.08).

**Table 5:** Correlation analysis of 13 quantitative traits of quince landraces used in the present study.

Traits	WFW	FH	10mm ABP	10 mm BAP	CH	CW	EW	MFL	SWT	SL	SW	SPF
FH	0.89**	1										
10mm ABP	0.90**	0.99**	1									
10mm BAP	0.89**	0.99**	0.99**	1								
CH	0.29	0.440	0.39	0.42	1							
CW	0.34	0.430	0.39	0.41	0.95**	1						
EW	0.83*	0.77*	0.76*	0.78*	0.30	0.41	1					
MFL	0.95**	0.93**	0.93**	0.93**	0.35	0.43	0.86*	1				
SWT	0.22	0.180	0.15	0.19	0.09	0.29	0.66	0.36	1			
SL	0.07	-0.01	-0.04	0.01	0.25	0.42	0.56	0.16	0.91**	1		
SW	0.19	-0.04	-0.06	-0.04	0.26	0.39	0.50	0.19	0.78*	0.87**	1	
SPF	0.04	-0.08	-0.10	-0.05	-0.18	-0.01	0.31	0.11	0.87*	0.69	0.64	1
MW	0.94**	0.80*	0.83*	0.80*	-0.07	0.01	0.72	0.86*	0.09	-0.11	0.02	-0.08

*. Correlation is significant at the 0.05 level.

**. Correlation is significant at the 0.01 level.

Note: WFW- Whole fruit weight, FL- Fruit length, ABP- Above basal point, BAP-below apical point, CL-Core length, CW- Core width, EW- Exocarp weight, MFL- Middle fruit length, SWT- Seed weight, SL- Seed length, SW- Seed width, SPF- Seeds per fruit, MW- Mesocarp weight.

### ANOVA analysis

For morphological variables with respect to area, there were significant differences among all most all variables (Table 6) except seed width. 10 variables (fruit weight, av. fruit width (10 mm above basal part), core length, exocarp) were highly significant with p<0.001. Two variables (core width and seed length) were found significant with p<0.05. Only seed width was insignificant with p>0.05. The variation within data values of all morphological variables were insignificant except Av. Fruit width which was least significant with p<0.05.

**Table 6:** Two-factor analysis of variance result for morphological traits and area.

Source	Dependent Variable	Sum of Squares	df	Mean Square	F	P
Area	Fruit weight	185772.50	6	30962.08	157.474***	0.00
	Fruit width	7212.86	6	1202.14	92.170***	0.00
	Core length	206.78	6	34.46	6.450***	0.00
	Exocarp	24.15	6	4.02	24.027***	0.00
	Seed weight	36.62	6	6.10	108.157***	0.00
	Seed width	8.17	6	1.36	2.466^ns^	0.05
	Mesocarp	242166.81	6	40361.13	189.287***	0.00
	Fruit length	7113.00	6	1185.50	89.134***	0.00
	Av. Fruit width	7128.24	6	1188.04	101.416***	0.00
	Core width	136.87	6	22.81	4.983*	0.00
	Av. Middle Fruit	5982.71	6	997.12	56.295***	0.00
	Seed length	20.68	6	3.44	2.618*	0.04
	No. of seeds	12394.57	6	2065.76	77.501***	0.00
Replication	Fruit weight	1811.50	4	452.87	2.303^ns^	0.08
	Fruit width	130.85	4	32.71	2.508^ns^	0.06
	Core length	31.62	4	7.90	1.480^ns^	0.24
	Exocarp	0.87	4	0.21	1.310^ns^	0.29
	Seed weight	0.62	4	0.15	2.788^ns^	0.04
	Seed width	0.74	4	0.18	0.336^ns^	0.85
	Mesocarp	538.54	4	134.63	0.631^ns^	0.64
	Fruit length	117.33	4	29.33	2.205^ns^	0.09
	Av. Fruit width	153.45	4	38.36	3.275*	0.02
	Core width	23.32	4	5.83	1.274^ns^	0.30
	Av. Middle Fruit	52.72	4	13.18	0.744ns	0.57
	Seed length	2.40	4	0.60	0.456^ns^	0.76
	No. of seeds	75.88	4	18.97	0.712^ns^	0.59

Note: ns (not significant)

‘***’ p<0.001

‘**’ p<0.01

‘*’p< 0.05

### Principal component analysis

The data obtained from principal component analysis was present in Table 7, with Eigen value of 0.48, with total variance of 97.78%. In the 1^st^ principal component, the total variation was 52.52%, where the contribution of all the quantitative traits was positively toward PC1. In PC2 the total variation was 80.15%, the contribution of Whole Fruit Weight, Fruit Length10 mm Below Basal Part, 10 mm Above Basal Part, and Mesocarp Weight was found positively while the Seed Weight, Seed Length, Seed Weight, Exocarp Weight, and Core Width was found negatively in PC2. Similarly, in PC3 the total variation was 94.06% and the contribution of different traits like Fruit Length, Core Length, Core Width, was found positively in PC3 while the remaining traits Mesocarp weight, Whole Fruit Weight, 10 mm Below Basal Part, and Exocarp Weight was found negatively contributed in PC3. In PC4 the total variation was 97.78%, where the contribution of Whole Fruit Weight, Exocarp Weight, Seed Weight and Mesocarp Weight was found positively toward PC4, while Fruit Length, 10 mm Below Basal Part, 10 mm Above Basal Part, Core Width, Middle Fruit length, and Seed Weight was found negatively toward PC4.

**Table 7:** Principal component analysis of 13 quantitative traits used in the present study of quince genotypes.

AXIS	PC1	PC2	PC3	PC4
Eigenvalue	6.83	3.59	1.81	0.48
% of Variance	52.52	27.63	13.90	3.72
Cum.% of Variance	52.52	80.15	94.06	97.78
Whole Fruit Weight	0.36	0.10	-0.11	0.33
Fruit Length	0.36	0.16	0.02	-0.24
10 mm above basal part	0.36	0.18	-0.01	-0.19
10 mm below apical part	0.36	0.15	0.00	-0.26
Core length	0.18	-0.05	0.65	0.00
Core Width	0.20	-0.14	0.59	-0.06
Exocarp weight	0.35	-0.14	-0.12	0.13
Av. Middle Fruit	0.37	0.05	-0.08	-0.04
Seed Weight	0.17	-0.45	-0.16	-0.25
Seed length	0.11	-0.49	0.04	0.10
Seed width	0.11	-0.46	0.02	0.53
Number of seed	0.05	-0.43	-0.29	-0.48
Mesocarp weight	0.31	0.18	-0.30	0.35

### DNA isolation, PCR amplification, and sequence analysis

The genomic DNA or gDNA of the collected genotypes of quince were isolated. Amplification of the gDNA with two gene loci- matK and ITS2 was carried out. Furthermore, 100% PCR amplification was shown by matK gene locus, while ITS2 gene showed 25% amplification success. That is why matK locus was used for this study. All the sequences were submitted to NCBI Genbank (Table 8). It was observed that matK region of Nalkot and Malam Jabba genotype showed 100% similarity with the reference sequences. Charbagh, Talash and Chitral genotypes showed 99% similarity with their reference sequences.

**Table 8:** Quince genotypes, using matK regions. Identification using BLAST and accession number.

S. No	Genotype	Species	Genomic region	BLAST similarity	Sequence Cover	E- value	Accession no.
1	Nalkot	*Cydonia oblonga*	matK	92.91%	100%	2e- 115	MN216014.1
2	Charbagh	*Cydonia oblonga*	matK	88.74%	99%	7e- 95	MN216014.1
3	Dardyal	*Cydonia oblonga*	matK	91.20%	95%	4e- 102	MN216014.1
4	Talash	*Cydonia oblonga*	matK	98.29%	99%	5e- 141	MN216014.1
5	Chitral	*Cydonia oblonga*	matK	91.50%	99%	3e- 108	MN216014.1
6	Tindodag	*Cydonia oblonga*	matK	92.47%	94%	1e- 106	KF861967.1
7	Malam jabba	*Cydonia oblonga*	matK	99%	100%	8e- 149	MN216014.1

### Phylogenetic analysis

**Fig 1:**
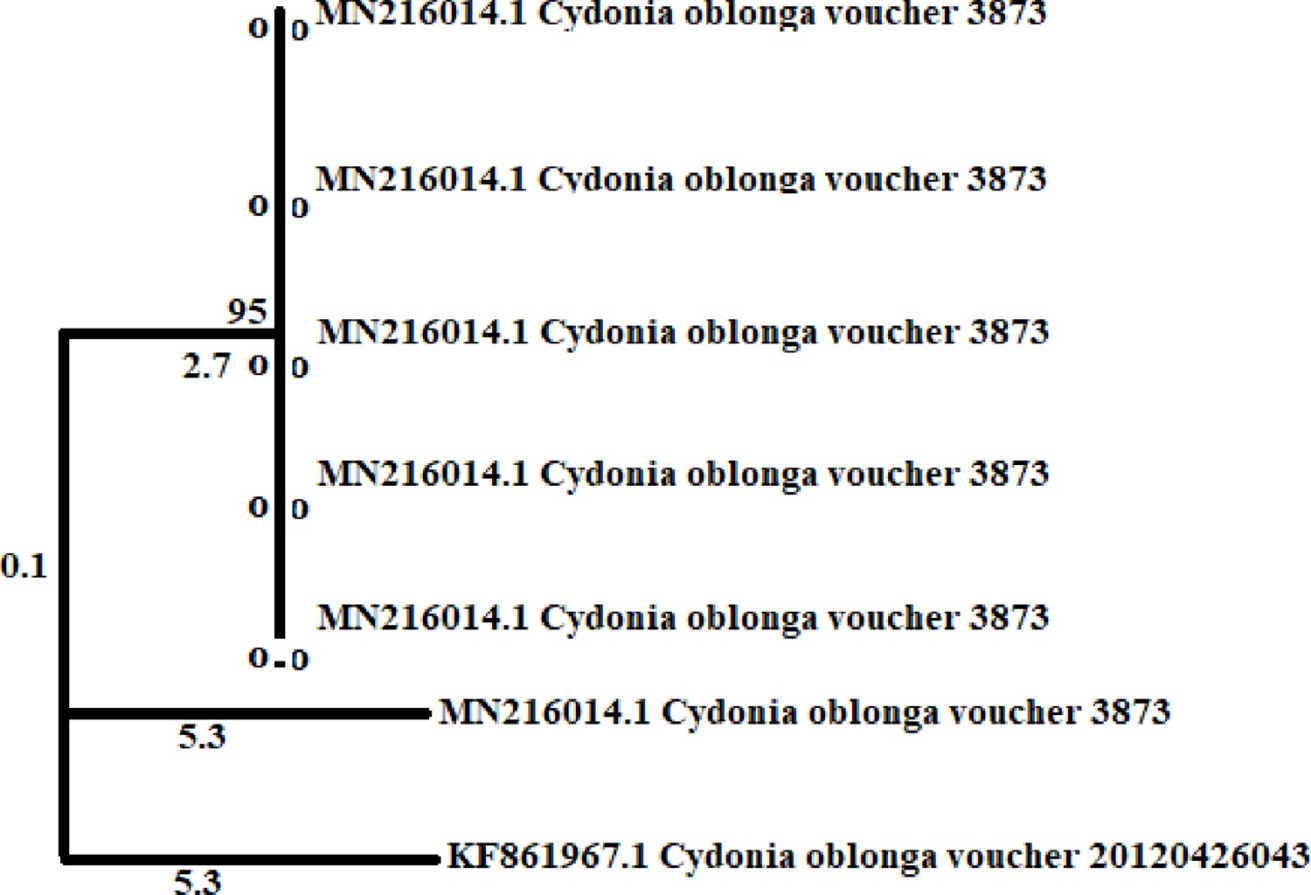
Phylogenetic relationship of quince (*Cydonia oblonga*) genotypes reported from Pakistan using DNA barcode locus i.e. matK region.

The phylogenetic relationship of Quince (*Cydonia oblonga*) genotypes was analyzed using one DNA barcode locus i.e. matK. Neighbor joining tree was constructed which was based on matK regions (Fig 1). It shows that the genotypes could be grouped into 3 main clusters. Nalkot, Charbagh, Talash, Dardyal and Chitral genotypes were grouped into one cluster. The tree shows that they are closely related as they show 95% similarity. Malam Jabba genotype was grouped into a separate cluster, though it had same accession number but its blast similarity and sequence cover separated it from the other cluster. Tindodag genotype was grouped into another cluster. Its accession number shows that it is completely new genotype of quince found in this region.

## Discussion

Plant morphology is one of the most important branch of plant sciences, besides plant genetics, plant physiology, taxonomy or plant systematics, environmental biology or plant ecology and evolutionary biology. However, these days, plant morphology is more like evo-devo (evolutionary and developmental biology) [16]. Evo- devo, actually, is composition of morphology and molecular genetics [17]. These days, plant breeders are interested in inter and intra population genetic variations. These inter and intra population variations is useful in analysing genetic structure of a germplasm [11]. Climatic conditions influence plant morphology which positively or negatively affect a given plant in an area [18].

In this study we focused on both qualitative and quantitative morphological variation of tree and fruit of Quince. But as multiple indicators such as climatic, topographic and edaphic factors have impact on phenotypic appearance [19], most of the qualitative morphological differences which are minute such as flowering time and ripening time may be neglected. Two qualitative traits which can easily distinguish a genotype we noted during our study were the taste and shape of fruit. Fruit of Tindodag genotype was reported as edible while all other fruits were astringic in taste. Chitral genotype’s fruit was Pyriformis in shape, while all other genotypes have maliformis shaped fruits. In the quantitative traits, whole fruit weight was noted between 68.38gm-328.82gm in all genotypes. Exocarp range between 2.49 and 5.16 gm, mesocarp ranged between 44.48 and 320.41gm. The results of Monka *et al*., [1] shows fruit weight of quince in range of 147.61–253.27gm, exocarp´s weight 28.50–43.89 gm, mesocarp´s weight 116.36–204.99gm. Rasheed *et al*., [20] found in his study that quince fruit average weight ranged between 40 to 234 grams. Further, they noted that some varieties may range between 300gm up to 2kg. Salaš [21] determines the weight of quince fruit on the basis of its variety and it ranges between 100 to 1200 grams. Comparing our results with the results of these authors we find some consistency with them. In this study we found average number of seeds range between 12.6 and 58.4 from genotype to genotype. Seed length range between 8.4 and 10.6mm while seed width ranges between 3.2 and 4.8mm. Monka *et al*., [1] noted number of seeds in quince fruit in range of 7 to 42. Further, the authors noted that seed length ranges between 6.7 and 9.65mm and seed width ranges between 4.41 and 5.52mm. Monka *et al*., [1] further added that seeds are very important resource because they contain up to 22% slime. The chemical composition of slime includes pentosan, carbohydrates, oil, and enzymes of cyanogenic glycoside amygdalin, which gives the typical fruit taste and aroma. Seeds of quince swells in water, and it is used both in medicines and cosmetics.

Phenotypic changes may be a result of environmental influence or strong genotypic heterogeneity [21, 22]. To confirm that the noted morphological changes are result of environmental factors and they are phenotypic changes only or there is also genetic diversity in these species, DNA Barcoding was done of all the genotypes. The BLAST results shows that all the genotypes have similar origin except Tindodag genotype which shows difference in its origin. Accession number for all other genotypes is MN216014.1, while accession number of Tindodag genotype is KF861967.1. It confirms that Tindodag genotype is not similar to other genotypes, which is also evident from morphological analysis because of fruit taste. As Tindodag genotype fruit is edible while all others are non-edible or astringic.

## Conclusion

Determination of genetic variation is very important among quince cultivars for breeding programs and management. Identification of quince genotypes using only morphological data is not sufficient in deciding a best cultivar. Use of molecular markers is the best solution for identification accessions at early stages. Different varieties of Quince were collected from different ecological zones of Khyber Pakhtunkhwa Pakistan among which 26 different morphological traits were targeted (13 qualitative and 13 quantitive). Tindodag variety was unique having heavy fruit weighing 328.82 g. The lowest fruit weight recorded was 68.38 g for Talash genotype. Charbagh and Tindodag genotypes seed were lengthy (10.6 mm) whereas Chitral variety has shortest seeds (8.4 mm). The coefficient of variance was ranged from 2.23% to 30.38%. Based on the study results, it can be concluded that quince plant is suitable for cultivation in this region and formers are encouraged to grow them in their field to meet the increasing demand of population as the plant has medicinal and commercial use. As the main focus of this study was to highlight suitable genotype for the region, it was noted that all genotypes were well adopted to the respective studied areas. As evident from morphological characters and DNA barcoding, all the genotypes have almost similar morphology (minute differences may be geographical or topographical), except Tindodag genotype. The uniqueness in Tindodag genotype is that it is edible and DNA barcoding confirms that its accession no. is separate from the rest genotypes.

### Permission and deposition of voucher

The ASRB University of Malakand approved the study whereas to preserve the specimen, a voucher specimen was deposited in the botanical garden University of Malakand, Pakistan with voucher no: UOM.Bot23/Garden 706. The plant was authenticated by Dr. Nasrullah Department of Botany, University of Malakand.

## References

[1] Monka A, Grygorieva O, Chlebo P, Brindza J. Morphological and antioxidant characteristics of quince (*Cydonia oblonga* Mill.) and chinese quince fruit (Pseudocydonia sinensis Schneid.). Potravinarstvo. 2014; 8. doi: 10.5219/415

[2] Ashraf MU, Muhammad G, Hussain MA, Bukhari SN. Medicinal Plant Rich in Phytonutrients for Pharmaceuticals. Frontiers in pharmacology. 2016; 7, 163. 27445806 10.3389/fphar.2016.00163 PMC4914572

[3] Raven PH. Flora of China. Beijing: Missouri Botanical Garden Press; 2013.

[4] Khoubnasabjafari M, Jouyban A. A review of phytochemistry and bioactivity of quince (*Cydonia oblonga* Mill.). Journal of Medicinal Plants Research. 2011; Vol. 5(16) pp. 3577–3594.

[5] Sajid SM, Zubair M, Waqas M, Nawaz M, Ahmad Z. A Review on Quince (*Cydonia oblonga*): A Useful Medicinal Plant. Global Veterinaria. 2015; 14 (4): 517–524.

[6] Rakesh S, Vinod J, Jai R. Nutritional composition and processed products of Quince (*Cydonia oblonga* Mill.). Indian Journal of Natural Products and Resources. 2011.

[7] Acikgoz C. Extraction and Characterization of Pectin Obtained from Quince Fruits (*Cydonia vulgaris* pers) Grown in Turkey. Asian Journal of Chemistry. 2010; 23. 149–152.

[8] Rop O, Balík J, Řezníček V, Juríková T, Škardová P, Salaš P, et al. Chemical characteristics of fruits of some selected quince (*Cydonia oblonga* Mill.) cultivars. Czech J. Food Sci. 2016; 29: 65–73.

[9] Tatari M, Abdollahi H. Evaluation of Vegetative and Reproductive Characteristics of Some Quince (*Cydonia Oblonga* Mill.) Genotypes from Central Regions of Iran. International journal of fruit Sciences. 2021.

[10] Bayazit S, Imrak B, Kuden A, Gungor MK. RAPD analysis of genetic relatedness among selected quince (*Cydonia oblonga* Mill.) accessions from different parts of Turkey. Hort. Sci. (Prague). 2011; 38:134–141.

[11] Ihsan M., Nazir N, Ghafoor A, Khalil A. A. K, Zahoor M, Nisar M, et al. (2021). Genetic Diversity in Local and Exotic *Avena sativa* L.(Oat) Germplasm Using Multivariate Analysis. *Agronomy*, 11(9), 1713.

[12] Kafkas S, Imrak B, Kafkas E, Sarıer A, Küden A. Quince (*Cydonia oblonga* Mill.) Breeding. 2018; Volume 3. 10.

[13] Nisar M, Khan A, Wadood SF, Shah AA, Hanci F. Molecular characterization of edible pea through EST-SSR markers. Turkish Journal of Botany. 2017; 41(4), 338–346.

[14] Arleo M, Ruibal F, Pereyra J, Miquel E, Fernández M, Martínez C. A DNA-based approach to discriminate between quince and apple in quince jams. International Food Research Journal. 2012; 19. 1471–1477.

[15] Arif IA, Khan HA, Bahkali AH, Al Homaidan AA, Al Farhan AH, Al-Sadoon M, et al. DNA marker technology for wildlife conservation. Saudi Journal of Biological Sciences. 2011;18 (3):219–225. doi: 10.1016/j.sjbs.2011.03.002 23961128 PMC3730548

[16] Sattler R, Rutishauser R. Fundamentals of Plant Morphology and Plant Evo-Devo (Evolutionary Developmental Morphology). Plants. 2023. 10.3390/plants12010118 PMC9823526 36616247

[17] Rutishauser R. EvoDevo: Past and Future of Continuum and Process Plant Morphology. Philosophies 2020; 5, 41.

[18] Nikoloudakis N, Bladenopoulos K, Katsiotis A. Structural patterns and genetic diversity among oat (Avena) landraces assessed by microsatellite markers and morphological analysis. Genet. Resour. Crop. Evol. 2016; 63, 801–811.

[19] Nadjat T, Sitayeb T. Effect of environmental conditions on morphological variability of leaves and fruits of five populations of Pistacia atlantica Desf. in North Algeria. Biodiversity Research and Conservation. 2020; 58. 1–12.

[20] Rasheed M, Hussain I, Rafiq S, Hayat I, Qayyum A, Ishaq S, et al. Chemical composition and antioxidant activity of quince fruit pulp collected from different locations. International Journal of Food Properties. 2018; 21. 2320–2327. doi: 10.1080/10942912.2018.151463

[21] Salaš P. Quince (*Cydonia oblonga* Mill.) and its growing and economic descriptions. [online], 2001; vol. 1, p. 3–7.

[22] Bonny SB, dJè y. Variabilité morphologique et agronomique des variétés traditionnelles de voandzou [*Vigna subterranea* (L.) Verdc. (Fabaceae)] de Côte d’Ivoire. J Appl Biosci. 2011; 41: 2820–2835.

